# Genomic structure and alterations of homeobox gene *CDX2* in colorectal carcinomas

**DOI:** 10.1038/sj.bjc.6690068

**Published:** 1999-02

**Authors:** O K Yagi, Y Akiyama, Y Yuasa

**Affiliations:** First Department of Surgery, Tokyo Medical and Dental University School of Medicine, 1-5-45 Yushima, Bunkyo-ku, Tokyo, 113-8519, Japan; Department of Hygiene and Oncology, Tokyo Medical and Dental University School of Medicine, 1-5-45 Yushima, Bunkyo-ku, Tokyo, 113-8519, Japan

**Keywords:** colorectal carcinoma, *caudal*-related homeobox gene, *CDX2*, mutation, loss of heterozygosity

## Abstract

Expression of *CDX2*, a *caudal*-related homeobox gene, was found to be decreased in colorectal carcinomas. Heterozygous null mutant mice as to *Cdx2* develop multiple intestinal adenomatous polyps. To clarify the role of *CDX2* in colorectal carcinogenesis, we determined its genomic structure, and searched for mutations of *CDX2* in 49 sporadic colorectal carcinomas and ten hereditary non-polyposis colorectal cancers (HNPCC) without microsatellite instability. None of them exhibited a mutation. We further examined 19 HNPCC carcinomas with microsatellite instability for mutations in a (G)7 repeat site within *CDX2*. One of them (5.3%) exhibited one G insertion. Loss of heterozygosity was observed in 2 of the 20 (10%) informative sporadic carcinomas, and in one of the three (33.3%) informative HNPCC cancers. These data indicate that *CDX2* may play only a minor role in colorectal carcinogenesis. © 1999 Cancer Research Campaign


*British Journal of Cancer* (1999) **79**(3/4), 440–444
© 1999 Cancer Research Campaign
Article no. bjoc.1998.0068

### Genomic structure and alterations of homeobox gene *CDX2* in colorectal carcinomas

**OK Yagi[1], Y Akiyama[2] and Y Yuasa[2]**

[1]First Department of Surgery and [2]Department of Hygiene and Oncology, Tokyo Medical and Dental University School of Medicine, 1-5-45 Yushima, Bunkyo-ku, Tokyo 113-8519, Japan

**Summary** Expression of *CDX2*, a *caudal*-related homeobox gene, was found to be decreased in colorectal carcinomas. Heterozygous null mutant mice as to *Cdx2* develop multiple intestinal adenomatous polyps. To clarify the role of *CDX2* in colorectal carcinogenesis, we determined its genomic structure, and searched for mutations of *CDX2* in 49 sporadic colorectal carcinomas and ten hereditary non-polyposis colorectal cancers (HNPCC) without microsatellite instability. None of them exhibited a mutation. We further examined 19 HNPCC carcinomas with microsatellite instability for mutations in a (G)7 repeat site within *CDX2*. One of them (5.3%) exhibited one G insertion. Loss of heterozygosity was observed in 2 of the 20 (10%) informative sporadic carcinomas, and in one of the three (33.3%) informative HNPCC cancers. These data indicate that *CDX2* may play only a minor role in colorectal carcinogenesis.

**Keywords:** colorectal carcinoma; *caudal*-related homeobox gene; *CDX2*; mutation; loss of heterozygosity

The homeobox is a highly conserved 180-bp DNA sequence encoding a 60-amino-acid motif termed homeodomain. The homeodomain acquires a helix–turn–helix structural conformation and is the sequence-specific DNA-binding domain of a family of transcriptional regulatory proteins (McGinnis et al, 1992). Genetic studies have clearly shown that homeobox-containing genes play fundamental developmental roles in determining the regionalization of body parts (McGinnis et al, 1992), organogenesis (Roberts et al, 1994), and the lineage of certain cell types (Li et al, 1990).

Human *CDX2* is a member of the *caudal*-related homeobox family, based on its high level of amino acid similarity within the homeodomain regions, and extensive conservation in the protein sequence outside the homeodomain of the *caudal* gene of *Drosophila melanogaster* (McGinnis et al, 1992). The *caudal* gene is necessary for anteroposterior polarity during early *Drosophila* development. *Caudal*-related genes of other species, including mouse, are also expressed early in embryogenesis. In mouse, Cdx2 is expressed extraembryonically at 3.5 days post coitum (d.p.c.) in the trophoectoderm, and later in some trophoectodermally derived placental tissues. Embryonic expression begins at 8.5 d.p.c. in the posterior gut, the tailbud, the posterior part of the neural tube, and the unsegmented paraxial mesoderm before the development of somites (Beck et al, 1995). In the later embryo and adult, Cdx2 expression is restricted to the intestinal epithelium, where transcript levels vary quantitatively along the rostrocaudal axis, expression being highest in the proximal colon (James et al, 1994).

*Received 2 March 1998*
*Revised 27 May 1998*
*Accepted 3 June 1998*

*Correspondence to:* Y Yuasa, Department of Hygiene and Oncology, Tokyo Medical and Dental University School of Medicine, 1-5-45 Yushima, Bunkyo-ku, Tokyo 113-8519, Japan

Importantly, Cdx2 protein expression is not confined to a particular cell lineage (James et al, 1994), suggesting that it may be responsible for establishing regional, rather than cellular, identity in the intestinal epithelium. Furthermore, as its expression is tissue specific and present from the early embryo to the adult (James et al, 1994), it is likely that *Cdx2* plays a role in both the establishment and maintenance of the intestinal epithelial phenotype.

Human *CDX2* has been localized to chromosome 13q12.3 (German et al, 1994). Cdx2 expression was examined in human and rat colonic neoplasms, and was found to be decreased in colorectal carcinomas (CRCs) (Ee et al, 1995; Mallo et al, 1997). In functional studies, *Cdx2* has been shown to be important in the regulation of intestinal gene transcription (Suh et al, 1994), and in the regulation of the differentiation and proliferation of intestinal cells (Suh et al, 1996). Heterozygous null mutant mice as to *Cdx2* develop multiple intestinal adenomatous polyps (Chawengsaksophak et al, 1997), strengthening the possible relationship between *Cdx2* and maintenance of the intestinal phenotype. However, it is uncertain whether or not the *CDX2* gene is mutated in CRC. To address this question, we searched for *CDX2* gene alterations in sporadic and hereditary non-polyposis colorectal cancer (HNPCC) CRCs.

### MATERIALS AND METHODS

### Subjects

A total of 49 tumours pathologically diagnosed as sporadic advanced CRC, without polyposis or a family history of HNPCC, and corresponding normal tissues were obtained by surgical or endoscopical resection. Twenty-nine CRCs were also obtained from Japanese patients that fulfilled the HNPCC criteria (Vasen et al, 1991; Muta et al, 1996) by means of surgical resection. Previous analysis of these samples demonstrated that 19 of them exhibited the microsatellite instability (MSI) phenotype. Genomic DNA was

**Table 1** The sequences of the primer sets for *CDX2* exons 1–3

| Primer set[a] | | Exon | Primer sequence[b] (5′–3′) | T (°C)[c] | Amplicon (bp) |
|---|---|---|---|---|---|
| 1 | U | 1 | AGCATGGTGAGGTCTGCTCC[d] | 62 | 161 |
| | D | | ACCGCCGTAGTCCGGGTACT | | |
| 2 | U | 1 | CAAGGACGTGAGCATGTACC | 58 | 219 |
| | D | | GCGTAGCCATTCCAGTCCTC | | |
| 3 | U | 1 | ATCCTGGCCGGCAGCGTATG | 60 | 168 |
| | D | | GGGTGGTGGTGCGGATGGTA | | |
| 4 | U | 1 | GGCCGCAGCCATGGGCTAC | 62 | 172 |
| | D | | GGGAGACAGCTGCTCGGCG | | |
| 5 | U | 1 | TGCTGCAAACGCTCAACCCC | 62 | 174 |
| | D | | ccttcccaagcaccctccga | | |
| 6 | U | 2 | ctgatgggctgccttgttgt | 58 | 153 |
| | D | | CTCCGGATGGTGATGTAGC | | |
| 7 | U | 2 | CGGCTGGAGCTGGAGAAGG | 64 | 148 |
| | D | | tggtcctctgccaggcagt | | |
| 8 | U | 3 | cacatcttcaccaccaccatgtgct | 60 | 178 |
| | D | | GACCTGGCTGAGGCTGGGGA | | |
| 9 | U | 3 | AAAATCAACAAGAAGAAGTTGC | 55 | 158 |
| | D | | GAGCCAGACACTGAGGCTT | | |
| 10 | U | 3 | GAAGTGTCCCAGAGCCCTTG | 60 | 156 |
| | D | | TCAGCCTGGAATTGCTCTGC[e] | | |

[a]U, sense primer; D, antisense primer. [b]Upper case letters correspond to exons, and lower case ones to introns. [c]T, annealing temperature. [d]Designed according to untranslated sequences upstream of the initiating ATG. [e]Designed according to untranslated sequences downstream of the stop codon.

extracted from frozen tissues or paraffin-embedded tissues as described previously (Blin et al, 1976; Goelz et al, 1985).

### Determination of the exon–intron organization

The genomic structure of *CDX2* was determined through the following steps. By comparing the mouse *Cdx2* genomic DNA (Genbank no. U00454) and human cDNA (Genbank no. Y13709) sequences, and making use of the high homology between these sequences, we could predict the possible splicing sites in the human *CDX2* genomic structure (Figure 1). Therefore, we designed two primer sets (for intron 1: 5′-gggctgctgcaaacgctcaacc-3′ and 5′-actcgatatttgtctttcgtcctgg-3′; and for intron 2: 5′-accaggacgaaagacaaatatcgag-3′ and 5′-acttctcagaggacctggctgag-3′) encompassing two candidate splicing sites from the human cDNA sequence, and used the human genomic DNA as a template. In addition, two other primer sets were used (primers 1U and 4D, and primers 9U and 10D in Table 1) to ensure that there was no unexpected splicing site. The genomic fragments encoding the human *CDX2* gene were obtained by two methods. First, standard polymerase chain reaction (PCR) was performed for 30 cycles at 94°C (1 min), 55–68°C (2 min), and 72°C (1 min), with a final 10-min elongation at 72°C. Second, long and accurate (LA)-PCR was performed when standard PCR failed to work. For this method, 30 cycles at 94°C (30 s) and 68°C (20 min), with final elongation at 72°C, were performed, using LA-*Taq* polymerase (Takara, Kyoto, Japan). The PCR products were then purified using a QIA-quick spin PCR purification kit (Qiagen, Chatsworth, CA, USA), and then sequenced directly with a cycle sequencing kit (TaKaRa, Kyoto, Japan) using end-labelled primers and the conditions specified by the manufacturer.

### PCR amplification of *CDX2* exons 1–3 and mutation screening by PCR-SSCP (single-strand conformation polymorphism)

According to the exon–intron boundary sequences we determined, ten sets of overlapping primers numbered 1–10 (5′ to 3′) were designed for all the three exons, including each splicing site of the *CDX2* gene (Figure 1, Table 1). Non-radioisotopic PCR-SSCP analysis was performed as described previously (Oto et al, 1993). Briefly, each PCR carried out comprised 30 cycles of denaturation for 1 min at 94°C, annealing for 2 min at 55–64°C and extension for 1 min at 72°C, followed by final extension for 10 min at 72°C. Dimethyl sulphoxide was added for all PCR at 5% (v/v). The PCR products and denaturing stop solution (95% formamide, 10 mM EDTA, 0.25% bromophenol blue and xylene cyanol FF) were heated at 80°C for 5 min and then cooled on ice rapidly, and then electrophoresed on non-denaturing 12.5–15% polyacrylamide gels containing 10% glycerol in Tris-glycine buffer (25 mM tris-HCl and 200 mM glycine, pH 8.3) using a commercially available minislab gel (90×70×1.0 mm) apparatus (Atto, Tokyo, Japan). The running conditions were 320 V for 2–4 h at 15°C. After electrophoresis, the gels were stained with silver (Dai-ichi, Tokyo, Japan). When abnormal patterns were observed on SSCP analysis, the PCR products were cloned into the pT7Blue(R)T-vector (Novagen, Madison, WI, USA) and sequenced as described above.

### LOH (loss of heterozygosity) analysis

LOH was analysed by comparing normal and carcinoma DNA by the PCR-SSCP method.

### RESULTS

### Structure of the *CDX2* genomic locus

The genomic structure of *CDX2* was determined as described in the Materials and Methods section (Figure 1). The sequencing revealed the sites and boundary sequences of each exon–intron junction. The human *CDX2* consists of three exons (541, 146 and 252 bp) separated by two introns of approximately 4.8 kb and 1.4 kb. This structure is similar to the mouse *Cdx2* gene (Genbank no. U00454), except for the shorter introns in the mouse gene. The primer set designed for each exon including exon–intron junctions is listed in Table 1.

### Mutations in *CDX2*

We did not observe any mutation in the entire *CDX2* gene in the 49 sporadic CRC or ten HNPCC CRC without MSI on PCR-SSCP analysis. Within the *CDX2* coding region, there is a seven-guanine repeat site eight codons away from the 3′ terminus. Thus, we further examined 19 HNPCC CRC exhibiting MSI in this region using primer set 10, and observed one G insertion in carcinoma 841 (Figures 2A and B). The alteration was not found in the normal cell DNA of this cancer patient, indicating a somatic mutation. This insertion results in a frameshift with a new stop codon located 72

*© Cancer Research Campaign 1999*

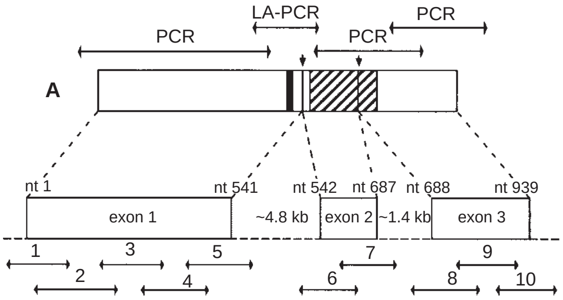

**Figure 1** Structure of human *CDX2.* (**A**) A schematic diagram of the organization of the human *CDX2* cDNA (Genbank no. Y13709), and the methods used for the isolation of genomic fragments. PCR, standard PCR; LA-PCR, long and accurate PCR. Arrowheads indicate the predicted splicing sites in the human *CDX2* cDNA, in comparison with the highly homologous mouse *Cdx2* genomic DNA (Genbank no. U00454). Homeodomain (cross-hatched region), a conserved sequence of 180 nucleotides shared by members of the homeobox gene family. Hexapeptide (solid region), a conserved hexapeptide motif upstream of the homeodomain, usually separated from the homeodomain by an intron, characteristic of the *caudal*-type homeobox genes (Suh et al, 1994). (**B**) Genomic structure of human *CDX2.* Boxes, exons; exon numbers in boxes, the numbers of the exons; -------, introns; kb, approximate lengths of the introns in kilobases; nt numbers above the boxes, nucleotide numbers corresponding to the *CDX2* cDNA sequence. The 5′ boundary of exon 1 is defined by the ATG start codon, and the 3′ boundary of exon 3 by the TGA termination codon. Also shown are the sites at which PCR products were generated by using the primer sets listed in Table 1 for mutation screening

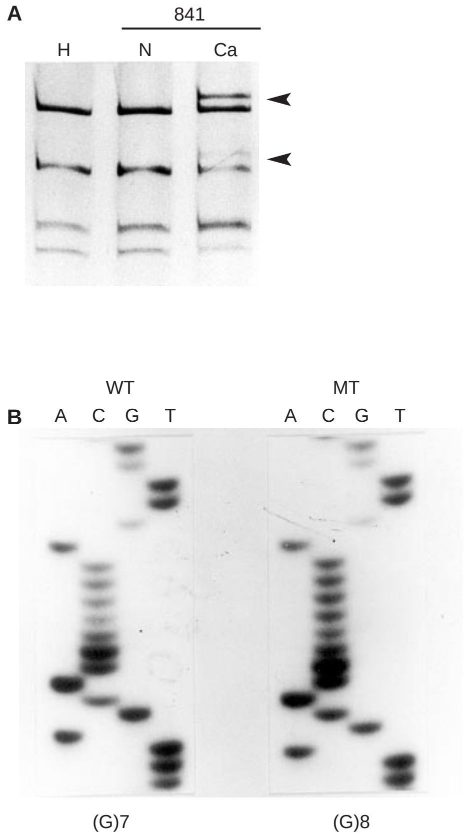

**Figure 2** Mutation analysis of exon 3 of the *CDX2* in HNPCC tumors. (**A**) SSCP analysis of the (G)7 repeat site by using primer set 10. The PCR products were electrophoresed in a 12.5% polyacrylamide gel. Healthy individual DNA (H) and normal tissue (N) both showed a normal SSCP homozygous pattern. Mobility shifts were detected in carcinoma 841 (Ca), as shown by arrowheads. (**B**) Sequencing analysis of the *CDX2* (G)7 repeat in carcinoma 841. The PCR products containing the (G)7 repeat site were cloned into a pT7Blue(R)T vector as described in the text and then sequenced. Mutant clones (MT) from carcinoma 841 showed eight guanines, (G)8, at the repeat site, whereas the corresponding normal tissue was (G)7 wild-type (WT)

nucleotides downstream. As a result, the COOH-terminal eight amino acids of CDX2 are substituted with 24 different amino acids, some encoded by nucleotides normally in the 3′ untranslated region. We examined the entire *CDX2* coding region in carcinoma 841, but we did not observe a second hit mutation.

### Polymorphic sites and LOH analysis

The PCR-SSCP pattern of normal cell DNA showed five sequence polymorphisms in primer sets 2, 5, 8–9 (overlapped), and 10 (two loci) (Table 2). Therefore, LOH analysis was carried out on these regions. The first variant, a CCG to CCC silent mutation, was detected in exon 1 codon 61. Heterozygosity was seen in 9 of the 49 (18.4%) sporadic CRC cases, and in two of the ten (20%) HNPCC cases without MSI. LOH was detected in two of the nine (22.2%) sporadic CRC informative cases, but in none of the HNPCC cases without MSI. Seven of the 48 (14.6%) healthy individuals examined exhibited such heterozygosity. The second variant, an AAC to AAG transversion in codon 164 of exon 1, changes asparagine to lysine. Only one of the 49 (2.0%) sporadic carcinoma samples showed heterozygosity at this site, however it did not exhibit LOH. Among the 48 healthy individuals examined, we found one (2.1%) individual exhibiting the same pattern.

The third variant, a CCG to CTG transition in codon 260 of exon 3, changes proline to leucine. This polymorphism was located in an area in which primer sets 8 and 9 overlapped, and therefore could be detected twice. Only 2 of the 49 (4.1%) sporadic carcinoma cases were heterozygous for this area, and

**Table 2** Polymorphic sites and LOH of *CDX 2* in sporadic and HNPCC CRC, and healthy individuals[a]

| | Codon 61 | | | Codon 164 | | | Codon 260 | | | Codon 293 | | |
|---|---|---|---|---|---|---|---|---|---|---|---|---|
| | Sporadic[b] | HNPCC[c] | Healthy[d] | Sporadic | HNPCC | Healthy | Sporadic | HNPCC | Healthy | Sporadic | HNPCC | Healthy |
| Homozygous A[e] | 39 (79.6) | 8 (80) | 41 (85.4) | 48 (98.0) | 10 (100) | 47 (97.9) | 47 (95.9) | 10 (100) | 47 (97.9) | 34 (69.4) | 9 (90) | 31 (64.6) |
| Heterozygous | 9 (18.4)[g] | 2 (20) | 7 (14.6) | 1 (2.0) | 0 (0) | 1 (2.1) | 2 (4.1) | 0 (0) | 1 (2.1) | 12 (24.5)[h] | 1 (10)[i] | 16 (33.3) |
| Homozygous B[f] | 1 (2.0) | 0 (0) | 0 (0) | 0 (0) | 0 (0) | 0 (0) | 0 (0) | 0 (0) | 0 (0) | 3 (6.1) | 0 (0) | 1 (2.1) |

[a]One healthy individual had a polymorphism in codon 310, an ACC to ACT silent mutation. [b]Sporadic, sporadic CRC (*n*=49). [c]HNPCC, HNPCC cases without MI (*n*=10). [d]Healthy, healthy individuals (*n*=48). [e]Homozygous A, G in codon 61, C in codons 164 and 260, and T in codon 293. [f]Homozygous B, C in codon 61, G in codon 164, T in codon 260, and C in codon 293. [g]LOH found in two of the nine (22.2%) informative cases. [h]LOH found in 1 of the 12 (8.3%) informative cases. It also exhibited LOH in codon 61. [i]LOH found in one of the one informative case.

 © Cancer Research Campaign 1999

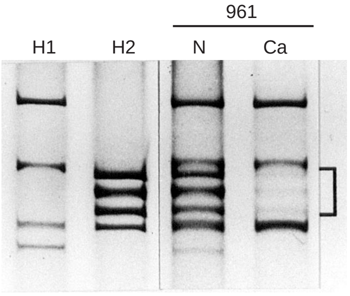

**Figure 3** LOH analysis of CRC. The PCR products of *CDX2* exon 3, using primer set 10, were electrophoresed on a 12.5% polyacrylamide gel. A decrease in the number of signals is seen in carcinoma 961 (Ca), compared with the heterozygous pattern of the corresponding normal mucosa (N). H1 and H2 are representative cases of the two types of homozygous pattern neither of them exhibited LOH. One of the 48 (2.1%) healthy individuals was heterozygous. The fourth variant, a TCT to CCT transition in codon 293 of exon 3 (Figure 3), changes serine to proline, and this heterozygous pattern was detected in 12 of the 49 (24.5%) sporadic carcinoma cases and in 16 of the 48 (33.3%) healthy individuals. One of the 12 (8.3%) informative cases exhibited LOH, and this was one of the two that exhibited LOH in codon 61. Moreover, one of the ten HNPCC cases without MSI was heterozygous and exhibited LOH at this site (Table 2). The fifth variant is a silent ACC to ACT transition in codon 310 of exon 3, which was detected in one of the healthy individuals. In carcinoma 841, the one exhibiting one G insertion at the (G)7 repeat site, a second hit inactivation by LOH could not be determined because it did not present any heterozygous pattern at these polymorphic sites. Combining all the polymorphic sites in *CDX2*, we found two LOH in a total of the 20 (10%) informative sporadic CRC and one LOH in the three (33.3%) informative HNPCC cases.

### DISCUSSION

Most sporadic CRC seem to undergo the traditional pathway of tumorigenesis (Kinzler et al, 1996), in which one observes mutations in *APC* for adenoma formation, K-*ras* for the adenomas to become larger, and *p53* for the carcinoma formation from these adenomas. In HNPCC, a so-called mutator pathway has been suggested, in which there is a germ-line mutation in one of the mismatch repair genes and a second hit in the wild-type allele would damage the DNA mismatch repair system (Hemminki et al, 1994). Through the resultant MSI phenotype, the *transforming growth factor β type II receptor* gene (A)10 and apoptosis-related *BAX* (G)8 repeat sites have been frequently found to be mutated in HNPCC carcinomas (Markowitz et al, 1995; Rampino et al, 1997; Yagi et al, 1998). Nevertheless, some types of CRC do not seem to undergo any of these two pathways (Yagi et al, 1997).

Embryological studies have supported the hypothesis that the *Cdx2* expression is tissue specific and present from the early embryo to the adult (James et al, 1994), therefore it is likely that Cdx2 plays a role in both the establishment and maintenance of the intestinal epithelial phenotype. Recently, the reduction in the mRNA and protein expression in CRC (Ee et al, 1995; Mallo et al, 1997), and the development of multiple intestinal adenomatous polyps in heterozygous null mutant mice as to *Cdx2* (Chawengsaksophak et al, 1997) have strengthened the correlation of this gene and the colorectal epithelium.

We analysed the entire coding region for *CDX2* in 49 sporadic advanced CRC and ten HNPCC cancers without MSI, but did not observe any mutation. However, when we examined 19 cases of HNPCC exhibiting MSI for a mutation in the seven-guanine repeat site, we observed a G somatic insertion in one case (carcinoma 841), it becoming G7/G8 heterozygous. This insertion results in a frameshift with a new stop codon located 16 amino acids downstream of the usual one. Carcinoma 841 belonged to a patient who had two synchronous CRC located in the transverse and descending colon respectively. In his family, there were eight cases of carcinomas related to HNPCC, most of which were CRC. We searched for second-hit inactivation of *CDX2* in carcinoma 841, however, no alteration such as a second-hit mutation or LOH was observed. Because no germ-line mutation was found in the HNPCC cases examined, a *CDX2* mutation may not be responsible for familial predisposition to cancer in HNPCC.

We observed five sequence polymorphisms in codons 61, 164, 260, 293 and 310. We did not find any discrepancy in the proportions of *CDX2* homozygous and heterozygous cases in all-type carcinomas compared with healthy individuals. For codon 61, two of the nine (22.2%) informative sporadic CRC exhibited LOH. For codon 293, 1 of the 12 (8.3%) informative sporadic carcinomas exhibited LOH, and this carcinoma was one of the two that exhibited LOH in codon 61 possibly indicating one whole *CDX2* allele deletion. For the remaining polymorphisms, there were only one or two informative cases. Totally, LOH was observed in 2 of the 20 (10%) and in one of the three (33.3%) informative cases in sporadic and HNPCC CRC respectively.

For codon 164, there was only one sporadic CRC case in which the sequence changed from AAC to AAG. Among the 48 healthy individuals examined, we observed such a pattern in one. Therefore, we considered it a polymorphism. However, this C to G transversion changes the predicted amino acid asparagine to lysine, and this C nucleotide is right at the 5′ extremity of the hexapeptide sequence which is a highly conserved domain and characteristic of the *caudal*-related family (Suh et al, 1994). Such a polymorphism might cause some change in the function of *CDX2*.

In conclusion, we found only one somatic mutation and three cases of LOH in *CDX2* in 78 CRC. Thus, we hypothesize that genetic alterations in the *CDX2* gene may play only a minor role in sporadic and HNPCC colorectal carcinogenesis. As for the down-regulation of the CDX2 mRNA and protein expression in CRC observed previously, further investigations, such as analysis of its regulatory mechanism, are necessary.

### ACKNOWLEDGEMENTS

This work was supported in part by a Grant-in-Aid for Scientific Research on Priority Areas from the Ministry of Education, Science, Sports and Culture of Japan, and by a Grant for Cancer Research from the Ministry of Health and Welfare of Japan.

### REFERENCES

Beck F, Erler T, Russell A and James R (1995) Expression of *Cdx-2* in the mouse embryo and placenta: possible role in patterning of the extra-embryonic membranes. *Developmental Dynamics* **204**: 219–227

Blin N and Stafford DW (1976) A general method for isolation of high molecular weight DNA from eukaryotes. *Nucleic Acids Res* **3**: 2303–2308

Chawengsaksophak K, James R, Hammond VE, Köntgen F and Beck F (1997) Homeosis and intestinal tumours in *Cdx2* mutant mice. *Nature* **386**: 84–87

© Cancer Research Campaign 1999 

Ee HC, Erler T, Bhathal PS, Young GP and James RJ (1995) Cdx-2 homeodomain protein expression in human and rat colorectal adenoma and carcinoma. *Am J Pathol* **147**: 586–592

German MS, Wang J, Fernald AA, Espinosa III R, Le Beau MM and Bell GI (1994) Localization of the genes encoding two transcription factors, *LMX1* and *CDX3*, regulating insulin gene expression to human chromosomes 1 and 13. *Genomics* **24**: 403–404

Goelz SE, Hamilton SR and Vogelstein B (1985) Purification of DNA from formaldehyde fixed and paraffin embedded human tissue. *Biochem Biophys Res Commun* **130**: 118–126

Hemminki A, Peltomäki P, Mecklin J-P, Järvinen H, Salovaara R, Nyström-Lahti M, de la Chapelle A and Aaltonen LA (1994) Loss of the wild type *MLHI* gene is a feature of hereditary nonpolyposis colorectal cancer. *Nature Genet* **8**: 405–410

James R, Erler T and Kazenwadel J (1994) Structure of the murine homeobox gene *Cdx-2*. Expression in embryonic and adult intestinal epithelium. *J Biol Chem* **269**: 15229–15237

Kinzler KW and Vogelstein B (1996) Lessons from hereditary colorectal cancer. *Cell* **87**: 159–170

Li S, Crenshaw III EB, Rawson EJ, Simmons DM, Swanson LW and Rosenfeld MG (1990) Dwarf locus mutants lacking three pituitary cell types result from mutations in the POU-domain gene pit-1. *Nature* **347**: 528–533

Mallo GV, Rechreche H, Frigerio J-M, Rocha D, Zweibaum A, Lacasa M, Jordan BR, Dusetti NJ, Dagorn J-C and Iovanna JL (1997) Molecular cloning, sequencing and expression of the mRNA encoding human Cdx1 and Cdx2 homeobox. Down-regulation of Cdx1 and Cdx2 mRNA expression during colorectal carcinogenesis. *Int J Cancer* **74**: 35–44

Markowitz S, Wang J, Myeroff L, Parsons R, Sun L, Lutterbaugh J, Fan RS, Zborowska E, Kinzler KW, Vogelstein B, Brattain M and Wilson JKV (1995) Inactivation of the type II TGF-β receptor in colon cancer cells with microsatellite instability. *Science* **268**: 1336–1338

McGinnis W and Krumlauf R (1992) Homeobox genes and axial patterning. *Cell* **68**: 283–302

Muta H, Noguchi M, Perucho M, Ushio K, Sugihara K, Ochiai A, Nawata H and Hirohashi S (1996) Clinical implications of microsatellite instability in colorectal cancers. *Cancer* **77**: 265–270

Oto M, Miyake S and Yuasa Y (1993) Optimization of nonradioisotopic single strand conformation polymorphism analysis with a conventional minislab gel electrophoresis apparatus. *Anal Biochem* **213**: 19–22

Rampino N, Yamamoto H, Ionov Y, Li Y, Sawai H, Reed JC and Perucho M (1997) Somatic frameshift mutations in the *BAX* gene in colon cancers of the microsatellite mutator phenotype. *Science* **275**: 967–969

Roberts CWM, Shutter JR and Korsmeyer SJ (1994) *Hox11* controls the genesis of the spleen. *Nature* **368**: 747–749

Suh E and Traber PG (1996) An intestine-specific homeobox gene regulates proliferation and differentiation. *Mol Cell Biol* **16**: 619–625

Suh E, Chen L, Taylor J and Traber PG (1994) A homeodomain protein related to caudal regulates intestine-specific gene transcription. *Mol Cell Biol* **14**: 7340–7351

Vasen HFA, Mecklin J-P, Meera Khan P and Lynch HT (1991) The international collaborative group on hereditary non-polyposis colorectal cancer (ICG-HNPCC). *Dis Colon Rectum* **34**: 424–425

Yagi OK, Akiyama Y, Ohkura Y, Ban S, Endo M, Saitoh K and Yuasa Y (1997) Analyses of the APC and *TGF-β type II receptor* genes, and microsatellite instability in mucosal colorectal carcinomas. *Jpn J Cancer Res* **88**: 718–724

Yagi OK, Akiyama Y, Nomizu T, Iwama T, Endo M and Yuasa Y (1998) Proapoptotic gene *BAX* is frequently mutated in hereditary nonpolyposis colorectal cancers but not in adenomas. *Gastroenterology* **114**: 268–274

 *© Cancer Research Campaign 1999*
